# State versus action orientation and compliance during the COVID-19 pandemic in Indonesia

**DOI:** 10.1016/j.heliyon.2022.e10725

**Published:** 2022-09-27

**Authors:** Andrie Elia, Danes Jaya Negara, Ferdinand, Sunaryo Neneng, Meitiana, Trecy Anden, Maria Haryulin Astuti, Hendrik Segah

**Affiliations:** a Department of Economics, Faculty of Economics and Business, University of Palangka Raya, Indonesia; b Department of Management, Faculty of Economics and Business, University of Palangka Raya, Indonesia; c Department of Animal Husbandry, Faculty of Agriculture, University of Palangka Raya, Indonesia; d Department of Forestry, Faculty of Agriculture, University of Palangka Raya, Indonesia

**Keywords:** Theory of planned behaviour, State orientation, Action orientation, COVID-19

## Abstract

This study explored the roles of activity versus state direction in how Indonesians adhered to COVID-19 preventive behaviour using the theory of planned behaviour (TPB). A total of 615 participants were gathered through a web questionnaire containing 68 questions. Structural equation modelling (SEM) was used to determine the causal relationships of the latent variable construct. The results revealed that information sources positively influence both attitudes and subjective norms toward preventive behaviour. The results also support self-regulation as a moderator of the main intent-behaviour relationship. Moreover, state-oriented people cannot self-regulate their behaviour to comply with COVID-19 protocols. This investigation provides productive experiences for strategists and managers to improve COVID-19 compliance. This paper's analysis also contributes to literature by revealing the intention to comply with COVID-19 protocols by emphasizing the role of self-regulation. The findings are relevant to practitioners and policymakers in the field of public health and managing of human behavior, particularly to improve of compliance with COVID-19 prevention. Therefore, governments or public health organizations can be utilized to improve the number of individuals who practice COVID-19 preventative behaviours now and in the future, but various factors must be addressed. Future directions and recommendations for improving TPB are suggested.

## 1 Introduction

As Indonesia is the fourth most populous country globally, it is predicted to experience more severe pandemic outcomes over time than other less populous countries (Djalante et al., 2020). However, when the SARS-CoV-2 novel coronavirus impacted China most severely from December 2019 to February 2020, Indonesia reported no cases of infection. Hence, Indonesian president Joko Widodo detailed the nation's to begin with two affirmed cases of COVID-19 disease on March 2, 2020; the government at the point went through ten months endeavouring to contain the infection. As of 10 November 2021, the Government of the Republic of Indonesia has reported 4.249.323 persons with confirmed COVID-19. There have been 143.592 deaths related to COVID-19 reported and 4.096.194 patients have recovered from the disease. WHO is working with the Indonesian Government to monitor the situation and prevent further spread of disease. Ultimately, Indonesia had the most COVID-19 cases in Southeast Asia (Tempo, 2022).

The greatest challenge confronted by the government intending to the widespread is peoples‘ a conduct (Bavel et al., 2020; Zacher and Rudolph, 2021). A survey conducted by the COVID-19 Handling Task Force indicates that people's compliance with “3M” behaviours—wearing masks, maintaining their distance, and washing hands—has decreased (Rumiati et al., 2021), although such behaviours are key to reducing infections. Regarding the public's compliance with COVID-19 prevention, several studies have observed two phenomena: the people who comply with COVID-19 mitigation (Briscese et al., 2020; Kuiper et al., 2020; Plohl and Musil, 2020), and those who refuse to comply with prevention measures (Nivette et al., 2021). For example, in the COVID-19 pandemic, vaccination beliefs and attitudes have been the most significant determinants of vaccination intention (Sherman et al., 2020). Positive attitudes toward epidemic prevention are drivers of preventive behaviour, and are linked to actual COVID-19 personal preventive measures (Andarge et al., 2020). Further, Plohl and Musil's (2020) global sample revealed that trust in science predicts compliance with COVID-19 measures; people with a greater trust in science exhibited greater compliance. Sailer et al. (2022) found that more scientific knowledge and trust in medicine in the United States affected individuals' behaviour, for example, in motivating people to socially distance. The apparent dread of COVID-19 infection is universally associated with expanded consistency.

Compliance also relates to the study of health behaviours, such as people's conduct while wearing a mask. Compliance is a positive behaviour demonstrated by society when people utilize face covers, socially distance, obey stay-at-home orders, shelter in place, self-quarantine or self-isolate, and avoid crowds, among various other strategies. Many factors influence compliance, including knowledge, motivation, perceptions, confidence in disease control and preventive activities, environmental variables, the quality of health education, and the capacity to use current resources (Yu et al., 2021). How the behavior of college students in Indonesia on COVID-19 pandemic? There is evidence that the prior research that have been described previously show that there is a significant relationship between student knowledge and awareness of the COVID-19 pandemic. The results of the research by Sailer et al. (2022) show that the knowledge variable has a significant influence on obedient behavior in using masks as an effort to prevent Covid-19 in Ngronggah, Kediri, East Java). However, the results of this study are not in line with research conducted by (Sutiningsih, 2021) and Kawareng et al. (2021) which showed that the respondent's behavior was good but not based on good knowledge about the prevention of a disease. Hence, the present study aims to explore compliance with COVID-19 measures and the role of self-regulation in moderating the effects of the intention-behaviour relationship.

## 2 Literature review and hypothesis development

### 2.1 Intention to comply with health behaviour

Health behaviours or health-related behaviours are individuals' actions that affect their fitness or humanity. These behaviours can be deliberate or inadvertent, and they can enhance or degrade an actor's or another person's health (Short and Mollborn, 2015). Furthermore, several acts may be categorized as health behaviours, such as smoking, drug use, nutrition, physical activity, sleep, hazardous sexual activities, health care in search of behaviours, and adherence to recommended therapeutic actions. Although health behaviours are commonly addressed at the individual level, they may also be assessed and summarized for people, groups, or communities (Chan et al., 2021). A sociological approach broadens the scope of such research by not only emphasizing the need to examine individual behaviours in context, but also acknowledging the significance of structure and agency. An earlier study used a socio-cognitive approach to explain compliance with health behaviours by utilizing Fishbein and Ajzen's (2011) theory of reasoned action factors, Bandura's self-efficacy variables, and the recurrence of brushing and flossing conduct (Tedesco et al., 1991).

Recently, Yastica et al. (2020) used the theory of planned behaviour (TPB) and the health belief model to distinguish the main drivers of unsatisfactory well-being conduct related to spreading sickness contamination. Their exploration was established depending on articles chosen that provided data concerning preventive well-being conduct, and particularly COVID-19 avoidance and the usage of the TPB and health belief model strategies.

These studies' outcomes demonstrated that people with greater anxiety about COVID-19 are more likely to positively change their behaviours, or specifically, to practice social distancing and improved handwashing. In contrast, an Italian study revealed that being negatively surprised by a hypothetical extension of such measures was associated with a lower willingness to comply (Briscese et al., 2020). Another investigation noted that non-adherence is higher among young adults who recently scored high marks for “solitary potential,” including low scores for the acknowledgment of “good” codes and exhibiting pre-pandemic lawful negativity, low disgrace or blame, low poise, high contribution to delinquent conduct, and relationships with misconduct and peers (Nivette et al., 2021). Young adults with low trust and who remembered the government's activities to battle the infection were likewise less consistent. These studies empirically prove that compliance with COVID-19 provisions depends on the people's level of fear of COVID-19; the individuals within this group also frequently do not consent to these arrangements.

### 2.2 The information source and its role

Health information-seeking behaviour is linked to epidemic information, which has long been used to explain behaviour (Brug et al., 2004; Higgins et al., 2011; Mertens et al., 2017). Health information-seeking is related to a range of characteristics. Search behaviour varies based on the type of information sought, the motivation to search, and the amount of experience (Gavgani et al., 2013). The information source can indicate one's capacity to recognize and comprehend a range of health information-seeking behaviours in the environment as well as any health hazards (Mertens et al., 2017). People also tend to search for information when their understanding of health and medical information is insufficient (Higgins et al., 2011). Individuals frequently assume that their perceived information search about a health danger, such as outbreaks of disease difficulties, is high when they believe they know more than others. According to existing research, health information-seeking behaviour, as a crucial cognitive component, is a significant driver of attitudinal and social variables in decision formulation and behaviour (Brug et al., 2004; Higgins et al., 2011). According to current research, females are almost sure to look for data on one's well-being; further, online customers who pursue well-being are better taught, procure more, and have more rapid Internet access at home and work (Rowley et al., 2017).

### 2.3 The moderating role of an action-state orientation

Although the TPB's capability has been demonstrated in various contexts (see Bin-Nashwan et al., 2021), previous research has revealed that the depth of its expectations for choices or practices requires an update by an expanding of its structure (Han et al., 2020). The TPB in particular has ignored the influence of self-regulation, which is thought to be critical in explaining health-promoting lifestyle habits, and especially self-regulation (Chen, 2017).

Many studies in existing literature, and notably in health behaviour, have demonstrated that self-regulation is a crucial notion to properly comprehend consumers' choice of forms and behaviours for both reliable and uncertain outbreaks. Such outbreaks and their social repercussions produce an abnormal condition, suggesting a low, predictable environment in which individuals cannot operate effectively for an extended period. One strategy for handling a low-predictability climate involves increasing resilience while replacing reactive behaviour with proactive activity to behave less emotionally and more confrontationally toward an unexpected circumstance (Pacholik-Żuromska, 2021). Models such as the TPB have provided a suitable forecast of people's intentions (the motivational phase), but only a reasonable prediction of their conduct (the intentional stage). According to Perugini and Bagozzi (2001), intention is a necessary but insufficient predictor of action. Thus, other factors are required to anticipate how individual act upon their eagerly, and hence, later hypothetical work has centered om the cognitive instruments by which eagerly are changed over into activity.

The propensity toward action versus a static orientation is one element that may impact the intention-behaviour connection. Kuhl (1992) created an activity-state direction to represent individual differences in volitional control limits in the design of self-rule. The resulting shape represents the capacity to begin and establish movement, manage time appropriately, handle interruptions, and work tirelessly on projects while overcoming setbacks and disappointment (Kuhl, 1985, 1992).

The action-state orientation is a dual concept, with action representing a higher self-regulation capacity and the state representing a lower self-regulation ability. Kuhl (1985) identified three types of action-state orientations: disengagement, initiative, and perseverance. People who are more activity-oriented can better avoid disconnection, or irrelevant thoughts; can exhibit initiative, or program and propose actions without difficulty; and persist in an activity over time, even when tempted by other external activities, more than individuals who are less action-oriented. More action-oriented individuals should exhibit better compliance with COVID-19 preventions throughout the pandemic because of the action-state orientation's connection with various advancement-related and goal-achieving behaviours.

It is also essential to assess the degree to which more significant action orientation levels might help students translate their attitudes and subjective norms into (a) intentions and (b) intentions to conduct. First, a person who is not action-oriented has a low propensity to act and plan, and thus, their attitude may not change to intent. A high action orientation offers a motivating propensity to initiate action, while attitude provides instructions and objects for actions (Bagozzi et al., 1992; Perugini and Bagozzi, 2001). As both are essential, the connection between attitude and the intention to comply should be more significant for those with higher rather than a lower action orientation. Second, subjective norms may assist the establishment of intentions in those who are not action-oriented. Individuals with a poor action orientation tend to absorb others' opinions, wants, and expectations are more significant for those with a low-action orientation (Kuhl, 1985). This implies that the link between subjective norms and intentions to comply is a high-action orientation. Kooij (2020) performed a theoretical assessment of self-regulation to examine how employees arranged their lives in connection with the COVID-19 epidemic. Finally, Bagozzi et al. (1992) speculated that the action-state orientation may be essential in converting intentions into actual conduct. This makes sense in the context of health behaviours and compliance with COVID-19 provisions in particular, as intentions may better predict health behaviour among individuals with high action orientation. This is often due to these individuals’ capacity to execute planning practice while avoiding both enthusiastic and physical diversions within the environment.

Various studies directly support the presumed link between action orientation and the regulation of emotion across a range of contexts (Backes et al., 2017; Bagozzi et al., 1992; Birk et al., 2020; Song et al., 2006). For example, Backes et al. (2017) illustrated that the relationship fulfilment among profoundly focused but action-oriented people and their accomplished is compromised by outside push less than that of state-oriented individuals and their partners. State-action orientation has also been shown to moderate the relationship between attitude and intention, and the relationship between intention and intensity (Song et al., 2006). In contrast, individual with a lower action orientation have less capacity to self-regulate, start goal-directed activities, and continue hindered assignments, indeed in the event that they are fair a tap absent (Birk et al., 2020). In particular, people who are more activity-oriented can better avoid irrelevant thoughts (disconnection), program and propose actions without difficulty (initiative), and persist on activity over time even when tempted by other external activities (persistence) than less action-oriented individuals. Individuals who are more action-oriented should also exhibit better levels of compliance with COVID-19 prevention measures throughout the pandemic because of the action-state orientation's connection with such behaviours as advancement and goal achievement.

This study investigates self-regulation, which is essential in explaining health-promoting lifestyle habits, as a significant mediator in the connection between intention and conduct. This finding suggests that the action–state orientation is crucial for converting intentions into actual behaviour.

### 2.4 The present study

**Figure 1:**
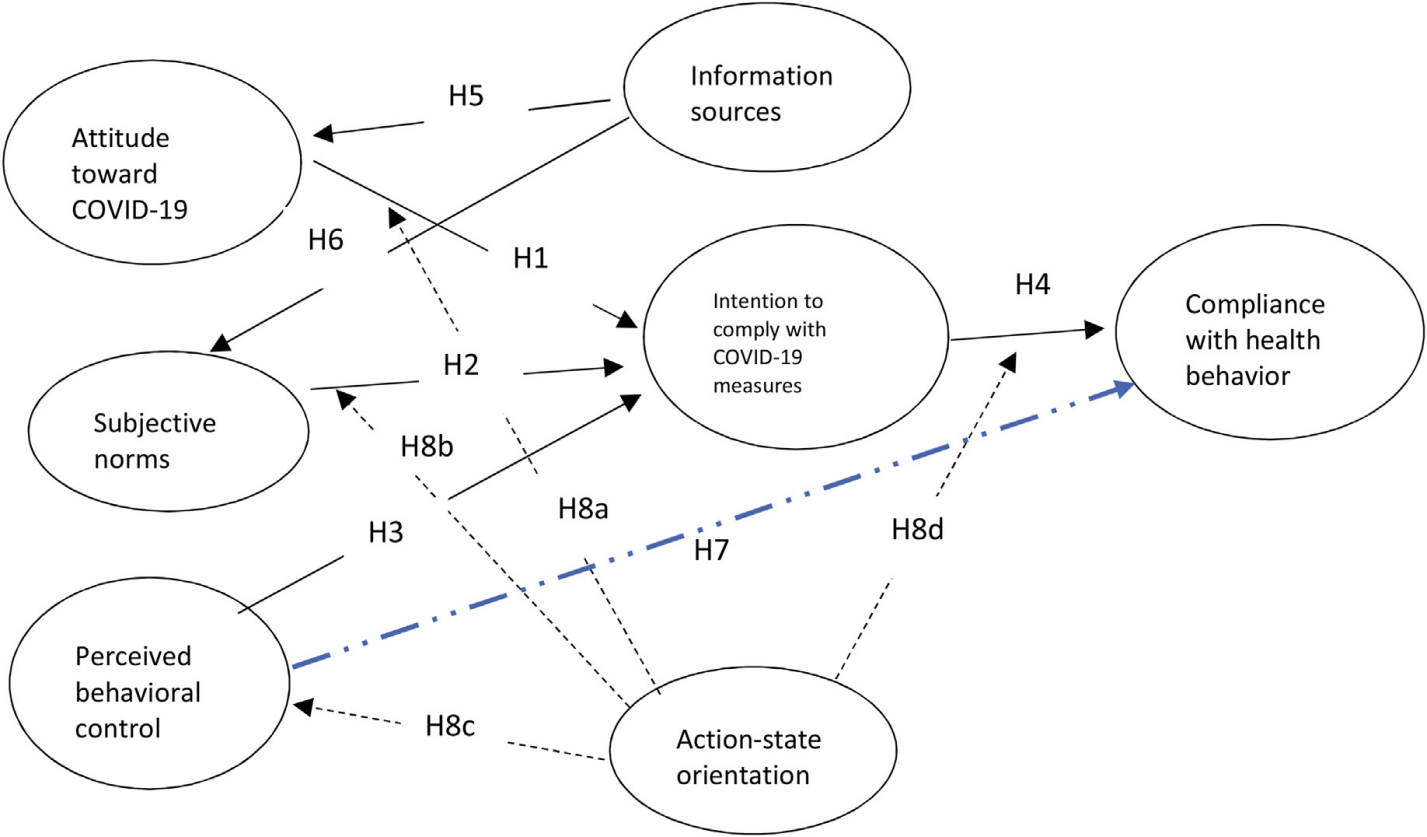
Proposed research framework.

The present study examines the intention to follow to COVID-19 preventive intentions and COVID-19 preventive behaviour within the framework of the theory of planned behaviour (Ajzen, 1991) to explain the action-state orientation's function. Specifically, we utilized the TPB model to examine the influence of the ongoing coronavirus epidemic in 2020 on Indonesian adherence to health behaviour. We also hypothesized that COVID-19 consumer health information-seeking behaviour can be the primary driver of subjective norms and attitudes, resulting in an approachable decision to comply with the provision. We also investigated support for the moderating effect of individual degrees of action versus the state orientation in the intention-behavioural relationship (Figure 1), as follows:

Hypothesis 1 “The attitude toward the behaviour positively affects the behavioural intentions for COVID-19 provisions among Indonesian students.”

Hypothesis 2 “The subjective norms positively affects the behavioural intentions for COVID-19 provisions among Indonesian students.”

Hypothesis 3 “Perceived behavioural control positively affects the behavioural intentions toward COVID-19 provisions among Indonesian students.”

Hypothesis 4 The intention to comply positively affects the

Hypothesis 5 “The COVID-19 information source positively affects Indonesian students' attitudes toward behaviours.”

Hypothesis 6 “The COVID-19 information source positively affects the subjective norms among Indonesian students.”

Hypothesis 7 *“*Perceived behavioural control positively affects the compliance with health protocol behavior.”

Hypothesis 8 The action-state orientation moderates the relationships between “(a) the attitude toward COVID-19 mitigation and the intention to adhere to COVID-19 mitigation measures; and (b) subjective norms and the intention to adhere COVID-19 mitigation measures.”

We anticipate that those who are more action-oriented demonstrate a stronger attitude-intention relationship than those who are less action-oriented. Further, those that are less action-oriented should establish stronger subjective norm-intention relationships than those that are more action-oriented:

**Hypothesis 8**: The action-state orientation moderates the relationship between the intention to comply and compliance with health behaviour.

## 3 Methodology

### 3.1 Participants and recruitment procedure

This paper provides explicit approval of the TPB structure using the sample of college undergraduates. The undergraduate student is a significant partner in the COVID-19 moderation issue for a few reasons.

The study from Barari et al. (2020) showed that seniors (60+), women, and healthier individuals said they were more compliant with most health practices than juniors (18–29). Meanwhile, the study of Nivette et al. (2021) showed that college students had higher non-adherence in young adults with high “independent potential”, while those with low affirmation of the prevailing code. Furthermore, a study by Roy-Chowdhury et al. (2020) reports that the age group of 25 and under is less worried about the coronavirus.

Furthermore, an analysis indicates non-adherence is higher in young adults with high “independent potential,” while those with a low affirmation of applicable codes, a natural wariness pre-pandemic, and low sense of shame/fault scored high for markers of “introverted potential.” Further, these latter individuals have an insufficient acknowledgment of relevant principles, lawful criticism pre-pandemic, a sense of shared disgrace/blame, low restraint, delinquent conduct, and relationships with wrongdoing and their peers (Nivette et al., 2021). Young adults with a humble sense of trust who consider the government's activities in battling the infection are also less consistent, and thus, they are appropriate as test subjects. These statement based on the studied such as Barari et al. (2020), Cohen et al. (2020), Roy-Chowdhury et al. (2020). Moreover, these opinions experimentally confirmed the fact that compliance with COVID-19 requirements is based on how persons who have a greater fear of COVID-19 and those who belong to the younger age group are the ones who frequently fail to assent to these arrangements (Nivette et al., 2021).

This examination gathered information along these lines by utilizing Google Docs or hangouts as an online platform for communication, rather than local research. Google's structural joints include posts and flows of different web-based media stages, such as WhatsApp groups and undergraduate Telegrams. The study's participants were educated onthe details of the examination's goals at the beginning of the study in gathering complete data, preserving respondent's confidentiality, and receiving consent from each respondent. The population of this study was composed of Indonesian undergraduate student aged 18–25 years living in central Indonesia territory (i.e. Kalimantan island). Only people living in Indonesia who were at least 18 years of age were allowed to voluntarily participate. According to this scenario, we conducted on Indonesian university students who life at central Indonesia territory. All sample respondents' identities were kept confidential to ensure their results could be utilized for research purposes. We previously conducted a pilot study with 80 individuals to test the underlying dependability of our estimations and to determine the nature of the survey. At that point, 729 Google invitations were sent for the initial overview, with 659 initial respondents.

### 3.2 Measures

A health behaviour assessment was adapted to fit the current study and determine compliance with COVID-19 mitigation strategies. Specifically, this study adapted particular questionnaires (Sallis et al., 2008; Zhang et al., 2020a, Zhang et al., 2020b) to assess respondents’ views of health behaviour, including the efficacy of COVID-19 preventive measures during a period of limited mobilization in Indonesia (see Table 1a and Table 1b).

**Table 1a:** The constructs and measurement items.

Construct	Items	Measures	Supporting References
Attitude	A1	I worry about the number of people infected by COVID -19.	Han et al. (2020 Lucarelli et al. (2020 Prasetyo et al. (2020) Nguyen et al. (2022)
	A2	I feel stressed during the COVID-19 outbreak.	
	A3	I am afraid that one of my family members will get infected.)	
	A4	I feel anxious during COVID -19 outbreak	
	A5	I feel insecure if someone stands too close to me during COVID -19 outbreak.	
	A6	I feel insecure if someone is not wearing a mask during the COVID-19 outbreak.	
	A7	I feel insecure if someone sneezes or coughs next to me.	
Subjective norm	NS1	Most individuals I know follow the government's preventative measures	Prasetyo et al. (2020) Yu et al. (2021) Zhang et al. (2020a) Andarge et al. (2020)
	NS2	Outside, the majority of the people I know wear face masks	
	NS3	Most people I know are staying at home and work from home.	
	NS4	Most people I know are using hand sanitizer	
	NS5	Most people I know, are doing physical distancing	Adam (2020)
Perceived Behavioral Control	PBC1	The preventive protocols are completely up to me	Prasetyo et al. (2020 Yu et al. (2021)
	PBC2	I think preventive protocols are easy to be implemented	
	PBC3	I am confident that I can prevent getting infected by COVID-19	
	PBC4	I am confident that I enough knowledge about COVID-19	
Information source	IS1	Have you read the health sections of a newspaper or a general magazine in the last 12 months?	Gavgani et al. (2013) Peng-Wei et al. (2020) Redmond et al. (2010)
	IS2	How frequently have you read such health sections in the previous year?	
	IS3	Would you say you read these health sections once a week, or more or less than once a week?	
	IS4	Have you seen any health features on the local news in the last 12 months?	
Intention	IF1	I am willing to take the cautioned precautions until the conclusion of the COVID-19 epidemic	Long and Khoi (2020)Nguyen et al. (2022)
	IF2	I am willing to obey every law hooked up by means of my government at some point of the COVID-19 outbreak	
	IF3	I am getting ready to observe my government's lead to impenetrable the country, city, and community	
	IF4	I am willing to follow my government to lock down the country, city, and community	
	IF5	I am willing to reschedule my travel plans	
Compliance Health Protocol Behavior	AB1	I am practicing proper handwashing to prevent the spread of the virus.	Andarge et al. (2020)Finlay et al. (2002)Lucarelli et al. (2020)Prasetyo et al. (2020)Yastica et al. (2020)
	AB2	Avoiding touching of eyes, nose and mouth	
	AB3	Covering mouth and nose when coughing or sneezing	
	AB4	Keep physical distancing	
	AB5	Self-isolate (not visit any family or friends)	
	AB6	Staying at home (sick or having cold)	
	AB7	Using face mask	
	AB8	I always dispose of my face mask properly	
	AB9	I keep working from home during the COVID-19 outbreak

**Table 1b:** Action control item^1^.

Construct	Items	Measures	Supporting References
Action Control	AS1	1. When I know I must finish something soon: A. I have to push myself to get started∗ B. I find it easy to get it done and over with	Backes et al. (2017)Diefendorff et al. (2000) Koole and Fockenberg (2011) Kuhl and Kazen (1994)
	AS2	2. When I don't have anything in particular to do and I am getting bored: A. I have trouble getting up enough energy to do anything at all B. I quickly find something to do∗	
	AS3	3. When I am getting ready to tackle a difficult problem: A. It feels like I am facing a big mountain that I don't think I can climb B. I look for a way that the problem can be approached in a suitable manner∗	
	AS4	4. When I have to solve a difficult problem: A. I usually don't have a problem getting started on it∗ B. I have trouble sorting out things in my head so that I can get down to working on the problem	
	AS5	5. When I have to make up my mind about what I am going to do when I get some unexpected free time: A. It takes me a long time to decide what I should do during this free time B. I can usually decide on something to do without having to think it over very much∗	
	AS6	6. When I have work to do at home: A. It is often hard for me to get the work done B. I usually get it done right away∗	
	AS7	7. When I have a lot of important things to do and they must all be done soon: A. I often don't know where to begin B. I find it easy to make a plan and stick with it∗	
	AS8	8. When there are two things that I really want to do, but I can't do both of them: A. I quickly begin one thing and forget about the other thing *I couldn*'t do∗ B. It's not easy for me to put the thing that *I couldn*'t do out of my mind	
	AS9	9. When I have to take care of something important but which is also unpleasant: A. I do it and get it over with∗ B. It can take a while before I can bring myself to do it	
	AS10	10. When I am facing a project that has to be done: A. I often spend too long thinking about where I should begin B. I don't have any problem getting started∗	
	AS11	11. When I have a boring assignment: A. I usually don't have any problem getting through it∗ B. I sometimes just can't get moving on it	
	AS12	12. When I have an obligation to do something that is boring and uninteresting: A. I do it and get it over with∗ B. It usually takes a while before I get around doing it	

1 Table 1b above is a list of action-control measure used to asses peoples' self-regulatory ability. Each item consist of an opening phrase and two alternative phrase and two alternative phrases that could be used to complete a sentence. One alternative indicates an action-oriented response; the other indicates a state-oriented response. Respondents selected the phrase best indicating their behaviour or thoughts in the situation describe.

#### 3.2.1 Variables of planned conduct

These items to measure intention to comply with COVID-19 mitigation measures include: “I am willing to take the cautioned precautions until the conclusion of the COVID-19 epidemic”; “I am willing to obey every law hooked up by means of my government at some point of the COVID-19 outbreak”; and “I am getting ready to observe my government's lead to impenetrable the country, city, and community.” The following items are used to assess subjective norms: “Most individuals I know follow the government's preventative measures” and” Outside, the majority of the people I know wear face masks. The alpha coefficient ranged from 0.727 to 0.848. All of the items in this study were graded on a five-point Likert scale, ranging from one “strongly disagree” to five “strongly agree.”

#### 3.2.2 Information sources

Information-seeking behaviour involves the deliberate search for information resulting from a desire to meet particular goals (Gavgani et al., 2013). Our survey asked about the resources and channels individuals use when seeking health information are questioned; respondents provided information regarding the resources and channels they used to gather health information, and how these were used with modifications (Gavgani et al., 2013; Redmond et al., 2010). The internet and other sources are used to get information about the coronavirus disease (Peng-Wei et al., 2020).

Respondents were given a list of five information resources and channels and asked to rate each one on a scale ranging from one (“never”) to five (“always”). The following are some examples of these items:

- “Have you read the health sections of a newspaper or a general magazine in the last 12 months?”
- “How frequently have you read such health sections in the previous year?”
- “Would you say you read these health sections once a week, or more or less than once a week?”
- “Have you seen any health features on the local news in the last 12 months?^1^”
- “How frequently have you seen health programs on local news in the previous year?”
- “Have you read such health information on the Internet in the last 12 months?”
- “Do any of these community organization(s) offer health information?”
- “How regularly do you discuss this health information with friends or family members?”

#### 3.2.3 Activity-state orientation

The activity-state orientation was evaluated using a modified Indonesian adaptation of the demand-related subscale of the ACS-90 (Kuhl and Kazen, 1994) and an updating model of volitional action control (Kuhl, 1985). The activity orientation was evaluated using a German adaptation of the Action Control Scale (Kuhl, 1992). The three measurements (distraction, dithering, and unpredictability) were surveyed with 12 items for each measurement. In each, individuals are presented with a circumstance (e.g., “When I need to tackle a troublesome issue…“), and need to choose one of two practices that were fairly valid for them. In the examination focusing on delays and distractions, state- and activity-oriented reactions were respectively scored as zero (e.g., “I consider different things first before beginning a job that needs to be done”) and one (e.g., “I begin et once”). Exploration of the activity orientation provided proof of the scale's legitimacy and dependability, including its factor construction and relationship with self-guidance (Koole and Fockenberg, 2011) given past research (Backes et al., 2017), as unpredictability illustrates the propensity to maintain one's conduct instead of actions to meet one's goals (control). Dependability in the state- and activity-oriented subscales is deemed acceptable with a Cronbach's α ≥ 0.70, as per past investigations (Diefendorff et al., 2000). “Action-oriented choices were coded as ‘‘1”, state-oriented choices were coded as ‘‘0” and summed for all 12 items.”

### 3.3 Ethical considerations

Ethical considerations in this study included obtaining approval from the ethics committee of Palangka Raya University, which funded this research, explaining the goals and working methods to the research participants and obtaining their consent, observing the principle of loss and the principle of confidentiality of the information, and omitting the names of the participants from the research instruments.

## 4 Data analysis and results

The data were analysed using Smart PLS 3.0 software, with partial least-squares structural equation modelling (PLS-SEM), which is divided into two stages (Hair et al., 2011). As Lucarelli et al. (2020) indicated, the PLS-SEM analysis begins with a focus on the reliability and validity of the measurements employed to represent each construct. Second, the theoretical model is evaluated to determine whether the assumptions are statistically supported (Jörg Henseler and Sarstedt, 2013). Validity and reliability are measured using a PLS analysis (Hair et al., 2011). At this stage, all assessments ensure that the tools used to measure concepts—for example, the attitude toward COVID-19 mitigation, subjective norms about COVID-19 comfort, perceived controls for COVID-19 convenience, the intention to comply with COVID-19 mitigation processes, and compliance with health behaviours—are both highly accurate and consistent (Hair et al., 2008).

### 4.1 Sample characteristics

All sample respondents’ identities were kept confidential to ensure their results could be utilized for research purposes. We previously conducted a pilot study with 80 individuals to test the underlying dependability of our estimations and to determine the nature of the survey. At that point, 729 Google invitations were sent for the initial overview, with 659 initial respondents. Of these, 627 finished the survey, and 615 substantial reactions were utilized for a final review of the information after omitting inadequate and inconsistent responses (Table 2). Each overview was finished in 12.8 min on average. Most participants (63.7%, *n* = 392) were female; 49.1% (*n* = 302) of the undergraduates revealed that web-based media and informal (public) websites were their primary source of COVID-19 data; 18.4% (*n* = 113) were senior students, or in at least their third year of undergraduate education; 34.9% (*n* = 215) had a COVID-19-infected family member or friend; and 65.5% (*n* = 403) revealed that their school furnished them with sufficient data about COVID-19 and how to manage it. Only a few of the undergraduates (25.7%, *n* = 158) reported that they had excellent experience in managing COVID-19.

**Table 2:** Respondents’ demographic information. Characteristics of some of the students who took part in the study (*n* = 615).

Characteristic	Number of students (%) *n* = 615
Mean age (SD)	22.6
Mean GPA	3.27
Gender	
Male	223 (36.3)
Female	392 (63.7)
Academic program	
Science	275 (44.7)
Humanities	340 (55.3)
Academic level	
1^st^ year	97 (15.4)
2^nd^ year	101 (16.5)
3^rd^ year	113 (18.4)
4^th^ year	105 (17.2)
5^th^ year	101 (16.6)
6^th^ year	98 (15.9)
Undergraduates' principal source of COVID-19 data	
Social media (Instagram, Telegram, Facebook, Twitter, WhatsApp, and unofficial websites)	302 (49.1)
Relatives, associates, and companions	156 (25.4)
Authorized site (the school or Ministry of Health)	122 (19.8)
Scientific catalogues and periodicals	35 (5.7)
Have any of your relatives or friends been infected with COVID-19?	
Yes	215 (34.9)
No	399 (65.1)
Did your school provide sufficient data about COVID-19 and how to manage it?	
Yes	403 (65.5)
No	212 (34.9)
On a scale of 1–10, indicate the degree to which you are content with your school's COVID-19 measures.	
1–3	111 (18.1)
4–7	390 (63.4)
8–10	114 (18.5)
Would you be interested in attending COVID-19's formal lectures?	
Yes	199 (32.4)
No	416 (67.6)
Gauge your readiness to face COVID-19 or a related pandemic.	
Not in any way ready	76 (12.4)
Fairly ready	381 (61.9)
Completely ready	158 (25.7)

### 4.2 Measurement model analysis

**Figure 2:**
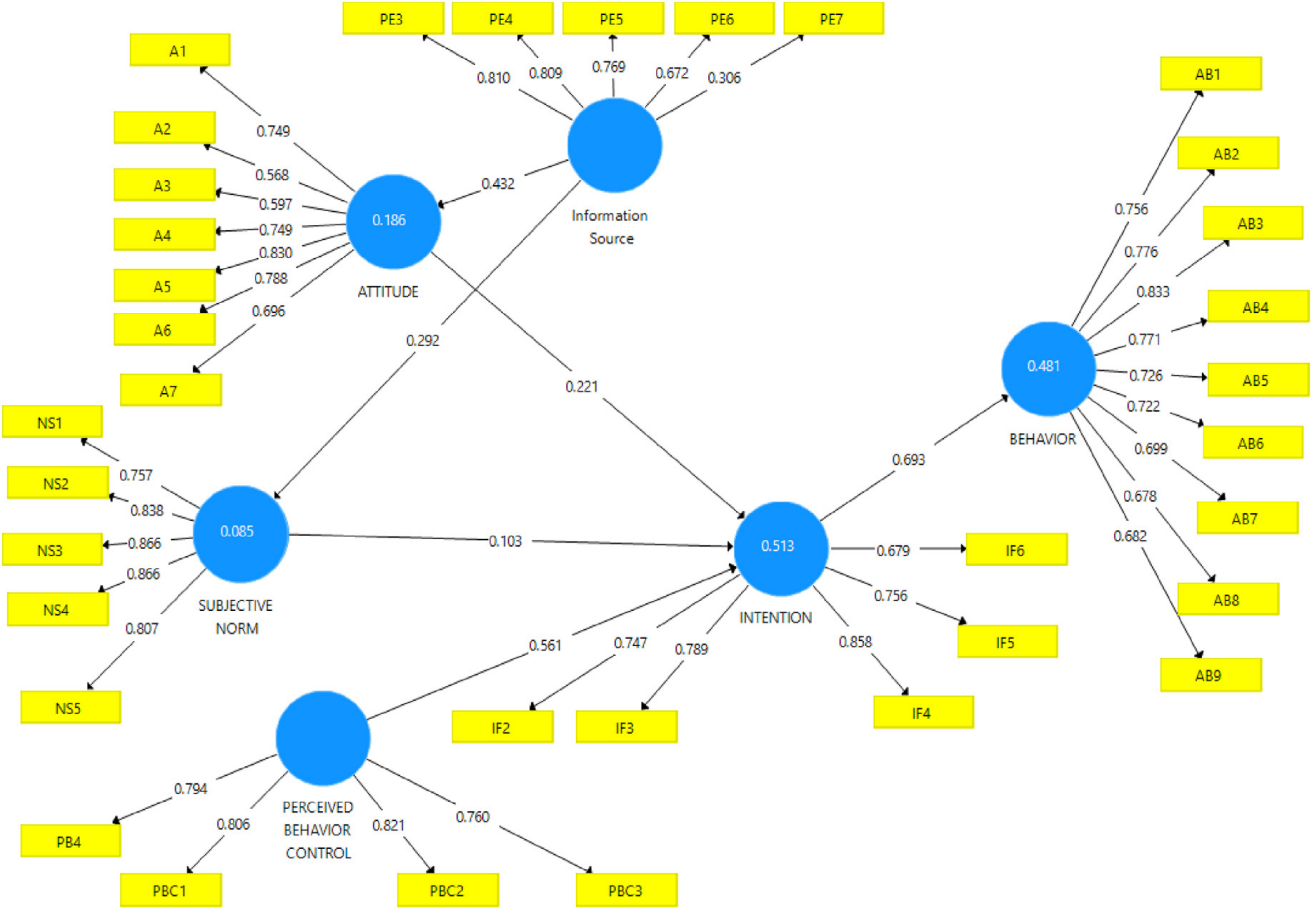
Measurement model (Partial least square (PLS)-algorithm).

**Figure 3:**
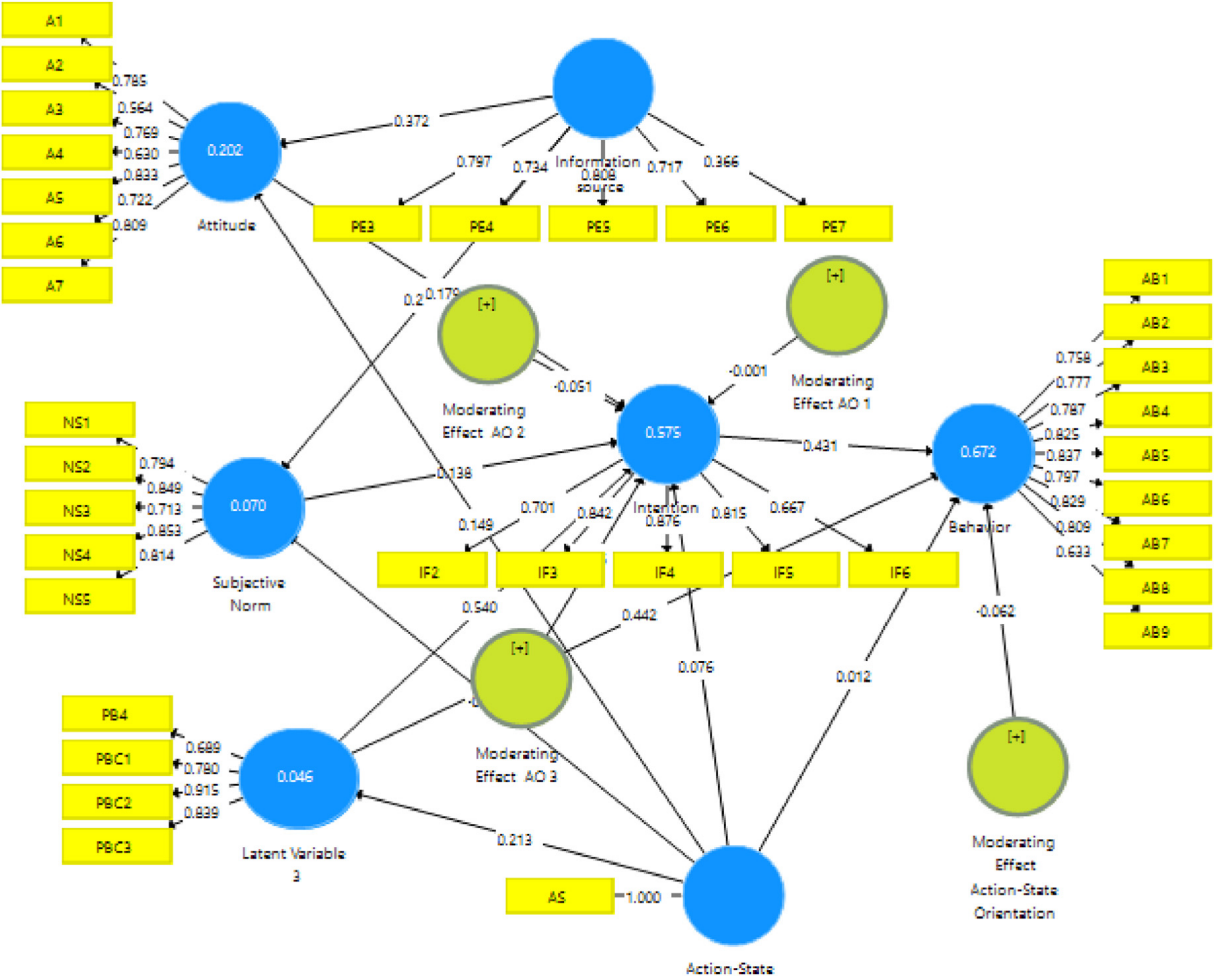
Moderating effect model.

Validity and reliability are measured using a PLS analysis (Hair et al., 2011). At this stage, all assessments aim to ensure that the tools used to measure concepts—for example, the attitude toward COVID-19 mitigation, subjective norms about COVID-19 comfort, perceived controls for COVID-19 convenience, the intention to comply with COVID-19, and compliance with health behaviours—are highly accurate and consistent (Hair et al., 2008). A PLS analysis indicates the outer loading for each construct (Hair et al., 2008), indicators of attitude, subjective norms, perceived behavioural controls, the information source, intention, and behaviour. The visible outside loading value of 29 was equal to or greater than 0.7. All indicators exhibited convergent validity for loading levels greater than 0.70. The findings demonstrate that all the constructions outperformed the specified values (see Figures 2 and 3).

The discriminant validity of variables was investigated using Heterotrait–Monotrait ratios (HTMT). Discriminant validity is confirmed by demonstrating that proportions of developments that must not be extraordinarily recognized with one another are not revealed to be profoundly linked with one another. It is the more resilient, superior, and influential approach, according to (Jörg Henseler et al., 2015), when compared to a standard (Fornell and Larcker, 1981). As given in Table 3, the conclusion shows that all HTMT values for the model successfully met the requirement proposed by (Kline, 2016), implying that the correlation between two concepts must be less than 0.9. Table 3 shows that the resulting value demonstrates adequate discriminant validity for the constructs used in this investigation. Discriminant validity is shown by presenting that proportions of developments that must not be extraordinarily recognized with one another are not revealed to be profoundly linked with one another.

**Table 3:** Discriminant validity.

	Attitude	HB	Information Source	Intention	PBC	SN
Attitude						
HB	0.758					
Information Source	0.532	0.513				
Intention	0.702	0.838	0.569			
PBC	0.752	0.778	0.384	0.762		
SN	0.416	0.504	0.295	0.489	0.491	

### 4.3 Structural model analysis

The structural model was evaluated by assessing the measurement model to test hypotheses H1–H8, structural equation modelling (SEM) was used to estimate the model shown in Figure 1. Table 4 presents the highest likelihood parameter estimates from the hypothesized model.

**Table 4:** Structural model assessment.

Hypothesis	Relationship	Path-Coefficient	Std. Error	*t*-value	*p*-value	Result	f2	R2	Q2	SRMR
H1	Attitude - > Intention	0.173	0.096	1.861	0.063	Not Accepted	0.387	0.106	0.331	0.08
H2	Subjective Norm - > Intention	0.138	0.033	4.243	0.000	accepted		0.044		
H3	PBC – > Intention	0.54	0.041	13.097	0	accepted	0.076	0.076		
H4	Intention - > Behavior	0.431	0.043	10.055	0	accepted	0.167	0.407		
H5	Information Source - > Attitude	0.372	0.041	9.159	0	accepted				
H6	Information source - > Subjective Norm	0.275	0.043	6.466	0	accepted				
H7	Perceived Behavioral Control - > Behavior	0.442	0.042	10.468	0	accepted				

#### 4.3.1 Hypothesis analysis

Except for attitude (H1), the data indicates that all hypotheses are supported because all route coefficients are significant (H2–H7), see Table 4. Moreover, the coefficient assessments regarding the attitude toward adhering to COVID-19 preventative procedures were in the proper direction. The subjective norm (0.138, *t* = 4.243 > 1.64, *p* < 0.05) and perceived behavioural control (0.54, *t* = 13.097 > 1.64, *p* < 0.05) significantly and positively impacted COVID-19 pre-emptive intentions*.* Similarly, perceived behavioural control (0.442, *t* = 13.097 > 1.64, *p* < 0.05) was associated with adherence to COVID-19 preventative measures; consequently, Hypotheses 2 and 3 were supported. Furthermore, adherence to COVID-19 preventive intentions (0.431, *t* = 10.055 > 1.64, *p* < 0.05) significantly and positively impacted the adherence to COVID-19 preventive behaviour.

We also evaluated the COVID-19 information source's projected effect (H5 – H6). The findings revealed that the information source significantly influenced attitudes toward preventive activity (0.331, *p* < 0.05) and the subjective norm for preventive conduct (0.235, *p* < 0.01). Thus, Hypotheses 5 and 6 were supported. In this study, we examined the impact moderator variable to the theoretical framework. The action-state orientation (−0.062, *t* = 2.487 > 1.64, *p* < 0.05) was also found to moderate the positive relationship between COVID-19 preventive intentions and COVID-19 preventive behaviour (H8). According to Henseler and Sarstedt (2013), the research model's quality is used to assess the dependent variables' predictive power. Model quality was measured, such as the significance of the path coefficient (*β* value), coefficient of determination (*R*^*2*^), predictive relevance (*Q*^*2*^), and effect size (*f*^*2*^). According to Hair et al. (2017), the *R*^*2*^ value for adhering to COVID-19 preventative behaviour is 0.672, indicating that the model's explanatory power is relatively robust. Similarly, *Q*^*2*^ is used to evaluate the model's predictive significance. A *Q*^*2*^ value greater than zero indicates that the model has predictive potential (Hair et al., 2017). The results reveal a *Q*^*2*^ value in the research model of 0.331, or the best predictive relevance.

As Cohen (1988) suggests, *f*^*2*^ scores of 0.02, 0.15, and 0.35 indicate modest, medium, and high impact sizes, respectively. In this model, the *f*^*2*^ scores support effect sizes ranging from modest to large. Table 4 displays the of *R*^*2*^, *Q*^*2*^, and *f*^*2*^ values. Furthermore, the model's overall fit—measured as the standardized root mean-square residual (SRMR)—was evaluated using the method developed by Henseler et al. (2016). The estimated findings in Table 3 indicate that the SRMR^2^ value (0.066) is a good match because it is less than 0.08, and this provides an appropriate fit (Jörg Henseler et al., 2016). Overall, the findings reveal that the information source, attitude, subjective norm, and perceived behaviour control substantially influence the adherence to COVID-19 preventative intentions. Such intentions also substantially and positively influence COVID-19 preventive actions.

#### 4.3.2 Multi-Group Analysis

We use Multi-Group Analysis (PLS-MGA) to test the possible moderating effect of action-state orientation in the TPB framework. Multi-group analysis is generally regarded as a special case of modelling continuous moderating effects (Henseler and Fassott, 2010). The analysis of moderator variable investigated the framework of how the role the variable state-action orientation to the variable attitude, subjective norm, and perceived behavioral control. The product indicator technique was used to examine the moderating influence of personality characteristics (Hair et al., 2008). A bootstrapping approach was used to determine if the interaction terms were significant. Table 5 displays the findings. The anticipated moderating impacts of the action-state orientation on the connections between attitude and intention, the subjective norm and intention, and perceived behavioural control and intention are addressed in Hypotheses 8a, b, and c, respectively. According to Table 5, self-regulation did not affect attitudes, subjective norms, or the PBC toward adhering to COVID-19 preventative measures. However, the coefficient calculations of the interaction terms for the subjective norm were correct, while the current findings indicate that the action orientation moderates the intention-behaviour relationships (−0.062, *t* = 2.487 > 1.64, *p* < 0.05). Thus, Hypothesis H8d is completely supported. The findings reveal a path strength from the action orientation that is greater than that of the state orientation. The action-state orientation's moderating impact increased the *R*^*2*^ value for this model from 0.536 to 0.672. Hence, we can infer that adding an action orientation improves the proposed model's explanatory capacity.

**Table 5:** Moderating effect of action-state orientation.

Moderating effect								
H8a	Moderating Effect AO 2 - > Intention	-0.051	0.029	1.791	0.074	Not Accepted		0.672
H8b	Moderating Effect AO 3 - > Intention	0.016	0.043	0.368	0.713	Not Accepted		
H8c	Moderating Effect AO 1 - > Intention	-0.001	0.019	0.054	0.957	Not Accepted		
H∗d	Moderating Effect Action-State Orientation - > Behavior	-0.062	0.025	2.487	0.013	Accepted	0.387	

## 5 Discussions

This study looked at how students acted in the face of the global pandemic of COVID-19. Our theoretical framework is based on the theory of planned behaviour to offer a clear understanding of people's behaviours toward COVID-19 pandemic decision-making processes, and specifically regarding their compliance with COVID-19 mitigation measures over which COVID-19 has comparatively less influence. The suggested model adequately enhanced the current social cognitive theory by considering the impacts of the COVID-19 information source and the action-state orientation. The model demonstrates how cognitive, volitional, and non-volitional dimensions can influence students' intentions to comply with health behaviour. The proposed model's components explained approximately 67.2% of the overall variance with actual behaviour. This number was higher than the initial theory of planned behaviour, which explained approximately 55.2% of the conflict in actual behaviour. Collectively, our theoretical framework is clearly useful in understanding students' types of choices.

Our findings revealed substantial connections between COVID-19 information sources and our variables of focus within the theory of planned behaviour. This finding suggests that COVID-19 public information sources in Indonesia helped strengthen the existing influence when social cognition theory predicted students’ post-pandemic intentions to comply with health regulations. Despite significant efforts regarding behavioural compliance (Nivette et al., 2021; Yu et al., 2021), this research is essential both theoretically and practically. Our findings provide critical information about the crucial role of perceived information searches regarding health threats, such as specific diseases, in elucidating their intention-generation process to comply with COVID-19 provisions. The current examination adds an acceptable imperative measurement to public conduct goal development, augmenting the current hypotheses in literature (Han et al., 2020).

Our analysis of the study variables' relevance revealed that the subjective norm contributes more to inducing Indonesian public behavioural intentions than other research constructs. This volitional component in particular was a significant predictor of post-pandemic behaviour to comply with COVID-19 mitigation strategies. This finding corroborated earlier studies that found the subjective norm necessary to explain one's safety or risk-based actions (Finlay et al., 2002; Prasetyo et al., 2020). This investigation provides valuable data, in that people's apparent prevailing burdens from family or companions, among others, are critical when settling on any choice connected to picking a safe social decision or facing challenges. From a theoretical perspective, our findings reveal the need to include this social dimension in a study framework concerning the safe choices among public risk-taking behaviours. From a practical perspective, dealing with subjective norms is an effective approach for the Indonesian government and its COVID-19 task force to improve citizens' post-pandemic compliance with COVID-19 mitigation measures. No evidence has been presented to indicate that personal characteristics cause individuals to differ regarding their behaviours or judgments when reacting to the environment, such as through compliance with COVID-19 mitigation.

In this study, self-regulation—essential to explaining health-promoting lifestyle habits—was investigated as a significant mediator in the connection between intention and conduct. This finding suggests that the action-state orientation is crucial in converting intentions into actual behaviour, as demonstrated by Backes et al. (2017). This is especially relevant in the health behavioural context: compliance with COVID-19 provisions may more highly predict health behaviour among those with a high rather than low action orientation. This is due to these people's capacity to carry out their plans while avoiding both emotional and physical distractions in their surroundings. This finding supports previous research, in that more action-oriented people can better prevent irrelevant thoughts. This process also includes planning and initiating actions without difficulty (initiative) and remaining with the activity over time, even when more interested in other external events (determination) than less action-oriented people (Diefendorff et al., 2000; Kuhl and Kazen, 1994).

However, only one interaction term was significant, indicating that the intention-behaviour relationship was stronger among state-oriented individuals. This study differs from others that have found no clear evidence for the moderating influence of state vs action orientation in the intention-behaviour link for certain behaviours.

Surprisingly, Song et al. (2006) discovered that an action-state orientation assists individuals in channelling intention into action. Individuals with a poor action orientation may intend to seek work, but they are less likely to channel this desire into action. Furthermore, Van Putten et al. (2010) discovered that persons with a state orientation tend to obsess over previous events and experience difficulty in forgetting them, and were especially vulnerable to the “sunk-cost” trap. The “sunk-cost” effect did not affect action-oriented people who could readily disregard previous experiences. Given the relatively minor influence of the state versus action orientation in intention-behaviour relationships, it may be essential to investigate conscious self-regulation (Morossanova, 2010).

## 6 Conclusion, limitations and implications

This study focused on student behavior and intention to compliance during the COVID-19 pandemic in Indonesia. The theory of planned behavior (TPB) construct had influenced the intention to compliance related to information source, state and action orientation (self-regulation) while taking a visit post-pandemic-19. Beyond the study, it is important to all health practitioners, universities, government and stakeholders might read this paper for recognition of the behavior and intention to compliance during covid-19 to prepare well.

### 6.1 Theoretical contribution

This study offers important to theoretical contributions to the current state of knowledge on people's perceptions of COVID-19 risk in Indonesia. The theoretical framework developed in this study enhances the TPB's predictive ability; it is generally relevant to people's perceptions of COVID-19 risk, and particularly when explaining self-regulation for public behavioural reactions. The suggested theoretical framework adds significant knowledge to the existing literature on the elements of disease outbreaks and general behaviour that are seldom covered in a solitary socio-psychological hypothesis. Generally, our hypothetical system is a crucial tool for better understanding the public's processing of COVID-19 information, with significant actions needed to protect themselves, and COVID-19 preventative behaviour providing safer destination choices. Consequently, the current study is valuable both conceptually and practically.

### 6.2 Practical implications

These results provide clear practical implications. Organizations and individuals can be utilized to improve the number of individuals who practice COVID-19 preventative behaviours now and in the future, but various factors must be addressed. As unanticipated in our data, the most relevant factors were perceived behavioural control and information sources. This study also indicates that people may or may not willingly adhere to COVID-19 preventions, requiring law enforcement's involvement (Chan et al., 2021). Governments or public health organizations should explore a non-coercive strategy aligned with core mental needs to foster an independent motivation to avoid COVID-19 infection.

While some networks are more conscious of the hazards than others, network responses should be critical in ending this epidemic. They should be viewed as part of the solution rather than the issue. During the early stages of the epidemic, it was clear that the government's scepticism and hesitancy regarding a possible pandemic in Indonesia, if not outright denial, had immediate consequences; one of these involved shifting populations away from a favourable pandemic risk assessment. This situation was accompanied by a relatively low knowledge of and drills for pandemics, although the Ministry of Health, among other organizations, facilitated such communications.

### 6.3 Limitation and future research

Despite the study's substantial offerings, we wanted to point out numerous limitations in addition to the study's noteworthy findings. First, our findings may be country-specific because our empirical data were generated from a sample of Indonesian college students. Future researchers could employ representatives from other nations to evaluate and extend this research through a sample with widely disparate viewpoints (Tejedor et al., 2021). However, the COVID-19 pandemic is a new worldwide threat, and thus, no scale has been established for comparison in this research. Although this study's questionnaire design was based on past research, we recognized several flaws. Given this, it is evident that future studies will deepen our understanding of the issue.

Second, past research suggests that action- or state-oriented persons provide little evidence for the moderating impact of the state versus action orientation. Meanwhile, it was shown to be significant in the current study, indicating that the intention-behaviour link was more critical among action-oriented persons. In considering the number of tests performed, it is also probable that this discovery might provide a favourable outcome from applying self-regulation to health behaviour. These findings are consistent with prior theoretical and empirical work highlighting the significance of research in the health sector, which is particularly scarce (Andarge et al., 2020; Kooij, 2020; Sailer et al., 2022). However, some studies failed to offer evidence for the moderating function of the state versus action orientation, even though a more comprehensive examination indicated that state-oriented persons were more likely to act on their intentions in the event of non-natively controlled behaviours (Norman et al., 2003). Future researchers might observe whether action-oriented employees are susceptible to the idea of trying (Bagozzi and Warshaw, 1990), which could explain such goal-seeking behaviour. According to Bagozzi et al. (1992), the theory of planned behaviour views action as a process or striving. The former regards action as a single performance of guided bodily movements, while the latter perceives action as one or more attempts, or a subsequent arrangement of endeavours toward a final presentation, with both physical and mental endeavours following the development of a goal to attempt.

In conclusion, we fostered a strong hypothetical structure regarding the intentions to adhere to COVID-19 preventive behaviour. This not only connects the COVID-19 information source to TPB focal constructs, but also incorporates an empirical approach to include the action-state orientation as a moderator. This study effectively included the critical elements in the reactions to COVID-19 in Indonesia and health behavioural settings in the suggested model by satisfying the essential requisites of the theory extension (Hagger, 2015). The integrated ideas conceptually differ from the critical components of the socio-psychological theory used in the current investigation.

## Declarations

### Author contribution statement

Danes Jaya Negara, Andrie Elia: Conceived and designed the experiments; Performed the experiments; Analyzed and interpreted the data; Contributed reagents, materials, analysis tools or data; Wrote the paper.

Sunaryo Neneng, Meitiana, Trecy Anden, Maria Haryulin Astuti, Hendrik Segah: Conceived and designed the experiments; Analyzed and interpreted the data; Contributed reagents, materials, analysis tools or data.

Ferdinand, Sunaryo Neneng, Meitiana, Hendrik Segah: Performed the experiments; Analyzed and interpreted the data; Contributed reagents, materials, analysis tools or data.

Danes Jaya Negara, Hendrik Segah: Analyzed and interpreted the data; Contributed reagents, materials, analysis tools or data; Wrote the paper.

### Funding statement

Dr DANES JAYA NEGARA was supported by Directorate of Research and Community Services, Universitas Palangka Raya [311/UN24.13/PL/2020].

### Data availability statement

Data associated with this study has been deposited at https://bit.ly/3vk31zD.

### Declaration of interests statement

The authors declare no conflict of interest.

### Additional information

No additional information is available for this paper.

## Footnotes

2 Note that the accuracy of the SRMR test of exact fit depends on the accuracy of the reference nomal distribution to the sampling distribution of the SRMR, and on the accuracy of the asymptotic approximation to the empirical mean and standard deviation of the sampling distribution of the SRMR. According to Pavlov et al. (2021) that the proposed reference normal distribution provides a good approximation to the sampling distribution of the SRMR when the model fits exactly, but additional statistical theory is needed to support the use of this reference distribution.

## Footnotes

1 We ask: Have you read such health information online in the last 12 months? It is meant that the internet is social media. According to the report (Stephanie, 2021), the average Indonesian spends 3 h and 14 min daily accessing social media. The covid-19 pandemic makes society rely more and more on the internet and spend more time in the virtual world. That is according to a report by the British media company. We Are Social. Social media users increased by more than **13 percent** over the past year, with nearly *half a billion* new users taking the global user total to almost **4.2 billion** by the start of 2021.
